# Phenolic and Carotenoid Profile of Lamb’s Lettuce and Improvement of the Bioactive Content by Preharvest Conditions

**DOI:** 10.3390/foods10010188

**Published:** 2021-01-18

**Authors:** Virginia Hernández, M. Ángeles Botella, Pilar Hellín, Juana Cava, Jose Fenoll, Teresa Mestre, Vicente Martínez, Pilar Flores

**Affiliations:** 1 Instituto Murciano de Investigación y Desarrollo Agrario (IMIDA), c/Mayor s/n, La Alberca, 30150 Murcia, Spain; virginia.hernandez5@carm.es (V.H.); mariap.hellin@carm.es (P.H.); juana.cava@carm.es (J.C.); jose.fenoll@carm.es (J.F.); 2 Departamento de Biología Aplicada, Escuela Politécnica Superior de Orihuela (EPSO), Universidad Miguel Hernández, Ctra de Beniel km 3.2, 03312 Orihuela, Spain; mangeles.botella@umh.es; 3 Centro de Edafología y Biología Aplicada del Segura (CEBAS), CSIC, Campus Universitario de Espinardo, 30100 Murcia, Spain; tmestre@cebas.csic.es (T.M.); vicente@cebas.csic.es (V.M.)

**Keywords:** corn salad, leafy vegetables, phytochemicals, liquid chromatography, mass spectrometry

## Abstract

This study characterizes the phenolic, carotenoid and chlorophyll profile of lamb’s lettuce, a vegetable whose consumption in salads and ready-to-eat products is constantly growing. The MS/MS analysis allowed the identification of thirty-five phenolic compounds including hydroxybenzoic and hydroxycinnamic acids, flavanones, flavanols and flavanones, many of which are reported here in lamb’s lettuce for the first time. Chlorogenic acid was the principal phenolic compound found (57.1% of the total phenolic concentration) followed by its isomer *cis*-5-caffeoylquinic. Other major phenolic compounds were also hydroxycinnamic acids (coumaroylquinic, dicaffeoylquinic and feruloylquinic acids) as well as the flavones luteolin-7-rutinoside, diosmetin-apiosylglucoside and diosmin. Regarding carotenoids, seven xanthophyll and four carotenes, among which *β*-carotene and lutein were the major compounds, were detected from their UV-Vis absorption spectrum. In addition, chlorophylls a and b, their isomers and derivatives (pheophytin) were identified. Preharvest factors such as reduced fertilization levels or salinity increased some secondary metabolites, highlighting the importance of these factors on the final nutritional value of plant foods. Lamb’s lettuce was seen to be a good potential source of bioactive compounds, and fertilization management might be considered a useful tool for increasing its nutritional interest.

## 1. Introduction

The regular consumption of fruit and vegetables has many benefits for human health in terms of reducing the possibility of developing chronic diseases [1]. It has been estimated that a high intake of fruit and vegetables can reduce the risk of developing cardiovascular diseases [2] and several types of cancer [3]. Most of these health-promoting effects are related to the vegetable bioactive content. Phenolics are the most abundant antioxidants in the human diet [4], of which approximately a third correspond to phenolic acids and two thirds to flavonoids [1]. Several reports indicate that polyphenolic compounds are effective in the prevention of diseases caused by long-term diabetes such as cardiovascular disease, neuropathy, nephropathy and retinopathy [5]. Moreover, phenolic compounds seem to inhibit cell proliferation and tumor metastasis and induce apoptosis in various types of cancer cells, including colon, lung, prostate, hepatocellular, breast cancer or multiple myeloma [6,7]. While many plants contain phenolic compounds, their concentration and chemical forms depend on individual plant species. Vegetables and fruits are also rich in carotenoids, molecules with a high antioxidant capacity, and the consumption of a diet rich in carotenoids is thought to reduce the risk of cancer, cardiovascular diseases, age-related maculopathy and cataracts [8]. The influence of chlorophylls on human health has not been as widely studied as that of phenolic compounds and carotenoids, although evidence of their benefits has been reported [9].

Due to their importance in human health, the characterization of bioactive compounds contained in fruits and vegetables and their related beneficial effects need to be studied in greater depth. Specifically, green leafy vegetables can be a source of ascorbic acid, flavonoids, phenolic acids and carotenoids, besides minerals, fiber and many trace elements. Lettuce and escarole have long been the most common vegetables used in salads, partly due to their healthy attributes, and several studies about characterization of polyphenols in lettuce can be found in the literature [10,11]. However, new leafy vegetables are increasingly consumed in salads, especially as ready to eat products. Among them lamb’s lettuce (*Valerianella locusta* L. Laterr.) has special relevance due to its pleasant taste and texture and nutritional value. However, information on *V. locusta* in the literature is scarce and mostly focuses on the shelf-life and quality changes that may take place during postharvest storage [12] or by some preharvest conditions [13,14,15,16]. Plant development and yield are strongly affected by mineral nutrition and environmental stresses that reallocate resources from primary to secondary metabolism with a direct effect on product quality [15]. In some species it has been shown that salt stress induced the synthesis of substances in proportion to the increase in NaCl concentrations, confirming the important role of these molecules for the tolerance to stress conditions in plants and salinity as an efficient technique for increasing the secondary metabolite content in plants [17]. However, little information on the phytochemical profile of *V. locusta* can be found, with the exception of some recent works [18]. Moreover, despite the well-documented impact that mineral nutrition and irrigation water quality have on the biochemical composition of plants, and hence on the nutritional value of vegetables, there is hardly any information about how these preharvest aspects can affect lamb’s lettuce quality.

The main objective of the present study was the characterization of the phenolic, carotenoid and chlorophyll profile of lamb’s lettuce. Taking into consideration mineral nutrition and salinity as two of the preharvest factors that most affect the quality of plant foods, the impact of fertilization and salinity (NaCl content) on the bioactive compound content of lamb’s lettuce was also evaluated.

## 2. Materials and Methods

### 2.1. Plant Material

Lamb’s lettuce (*Valerianella locusta* L. Laterr. cv. Favor) plants were grown in a greenhouse equipped with a dynamic root floating system that pumped the nutrient solution from a tank into different containers (trays). Plants were supported through a floating board made of high-density polyethylene. The roots were fully submerged in the nutrient solution that circulated back to the tank through a drain for reuse. The pH of each nutrient solution was adjusted to between 5.5 and 6.0 every day. Water lost by transpiration was replaced every two days and nutrients were added every week to restore their initial concentrations. In order to study the impact of fertilization and salinity on the bioactive compound content, the control nutrient solution [½ Hoagland solution, electrical conductivity (EC)1 dS cm^−1^, 7 mM N, 2 mM Ca and 3.5 mM K] was modified to obtain different treatments in four consecutive experiments with different levels of nitrogen (0.1, 1 and 7 mM N), calcium (0.5, 2, and 5 mM Ca), potassium (0.1, 0.5 and 3.5 mM K), and salinity (0, 15, 30 and 60 mM NaCl), respectively. In every experiment, the plants were distributed in two blocks with three replicates per treatment and block. Each replicate consisted of a tray (3.6 m^−2^) containing 100 plants m^−2^. Salinity treatments consisted of applying 15 mM NaCl on one, two or four days (for the 15, 30 and 60 mM NaCl treatments, respectively) in order to avoid an osmotic shock. Final ECs of the different saline treatments were 1 (control), 2.7, 4.0 and 6.5 dS cm^−1^. Thirty days after transplanting (DAT), when the plant had five fully expanded leaves, fifty plants per replicate were harvested and weighed after being washed and gently dried. They were then powdered with liquid N_2_ and kept at −80 °C until subsequent analysis. Each sample was analyzed in triplicate.

### 2.2. Metabolite Analyses

#### 2.2.1. Phenolic Compounds

Phenolic compounds were extracted with methanol:formic acid (97:3) according to Cantos et al. [19] and analyzed using an Agilent 1200 liquid chromatograph (Santa Clara, CA, USA) equipped with a G6410A triple quadrupole mass spectrometer detector (MS/MS) equipped with an electrospray ionization (ESI) interface, operating in negative ion mode. A Lichrosphere C_18_ analytical column of 250 mm × 4 mm and 5 µm particle size was used (Agilent Technologies, Waldbronn, Germany). The mobile phase was 0.1% formic acid in water (solvent A) and 0.1% formic acid in acetonitrile (solvent B) at a flow rate of 1 mL·min^−1^. The gradient began with 5% B, raised to 10% B in 9 min, 30% in 50 min, and 100% in 2 min and held at 100% B for an additional 3 min before returning to initial conditions in 1 min and remaining isocratic for 6 min. The following operation parameters were used: 2000 V capillary voltage, 60 psi nebulizer pressure, 13 L/min drying gas flow and 350 °C drying gas temperature. Fragmentor voltages (F) from 20 to 200 V and collision energies (CE) from 2 to 50 V were used for optimizing selective reaction monitoring (SRM) transitions. Myricetin (Sigma-Aldrich, St. Louis, MO, USA) was used as internal standard. The phenolic compounds were identified by MS/MS experiments: full scan and neutral loss (NL), precursor ion (PreI) and product ion (ProdI) scan modes. Protocatechuic, luteolin−7*-O-*glucoside, diosmetin, diosmin, apigenin−7*-O-*glucoside, hesperidin (hesperetin−7*-O-*rutinoside) (Extrasynthese, Genay, France) and chlorogenic acid (5*-O-*caffeoylquinic acid), caffeic acid, *p-*coumaric acid, luteolin, and quercetin were quantified with respect to their standards (Sigma-Aldrich, Steinheim, Germany). Chlorogenic isomers were quantified with respect to chlorogenic acid; caffeic acid*-O-*hexosides, dicaffeoylquinic and caffeoylferuloylquinic acid with respect to caffeic acid; sinapic-hexose with respect to sinapic acid (Sigma-Aldrich, Steinheim, Germany); coumaroylquinic isomers with respect to *p-*coumaric acid; feruloylquinic isomers with respect to ferulic acid (Sigma-Aldrich, Steinheim, Germany); isorhamnetin-rutinoside with respect to isorhamnetin (Sigma-Aldrich, Steinheim, Germany); luteolin-apiosylglucoside and luteolin−7–rutinoside with respect to lutein-7*-O-*glucoside; quercetin-glucuronide, quercetin-glucoside with respect to quercetin; apigenin-rutinoside and acacetin-rutinoside with respect to apigenin−7*-O-*glucoside; diosmetin-apiosylglucoside with respect to diosmin.

#### 2.2.2. Carotenoids

Carotenoids and chlorophylls were extracted with methanol/tetrahydrofuran (1:1, *v*/*v*) containing MgO (Merck, Darmstadt, Germany) and 0.1% (*w*/*v*) butylated hydroxytoluene (BHT) (Sigma-Aldrich, St. Louis, MO, USA) following the methodology validated by Motilva et al. [20]. For that, an Agilent Series 1100 liquid chromatograph (Santa Clara, CA, USA) equipped with a photodiode array detector (DAD) and a 250 mm × 4.6 mm i.d., 3 μm two serially coupled Prontosil C_30_ columns Bischoff (Leonberg, Germany) were used. The mobile phase was methanol (solvent A) and methyl tert-butyl ether (solvent B) eluted at a flow rate of 1.0 mL/min, as follows: (1) initial conditions 15% solvent B and 85% solvent A, maintained for 20 min (2) a 20-min linear gradient to 30% solvent B, then maintained for 10 min (3) a 80-min linear gradient to 90% solvent B. Compounds were eluted and recorded for 70 min and the subsequent gradient allowed the column to be cleaned. All-*trans*-violaxanthin, 9 *cis*-neoxanthin, antheraxanthin, all-*trans*-lutein, zeaxanthin, *β*-cryptoxanthin, all-*trans*-*β*-carotene and all-*trans*-α-carotene were quantified using commercially available external standards (DHI LAB, Hoersholm, Denmark). Luteoxanthin was quantified with respect to antheraxanthin. The *cis* isomers of *β*-carotene were quantified with respect to all-*trans*-*β*-carotene. *β*-apo-8′-carotenal (Sigma-Aldrich, St. Louis, MO, USA) was added as internal standard.

#### 2.2.3. Vitamin C

For the study of the impact of fertilization and salinity on the bioactive composition, the vitamin C concentration was determined according to Fenoll et al. [21], using HPLC with an MS/MS detector.

#### 2.2.4. Statistical Analysis

The results were statistically analyzed using IBM SPSS Statistic 21 by analysis of variance (ANOVA) and Tukey’s test for differences between means.

## 3. Results and Discussion

### 3.1. Phenolic Compounds

#### 3.1.1. Hydroxybenzoic Acid

Protocatechuic acid (compound **1**) was directly identified by comparing its retention time and mass spectrum with those of the corresponding standard, with [M − H]^−^ at m/z 153 and a main fragment at m/z 109 due to the loss of CO_2_ from the carboxylic acid [22] (Table 1). It is a widely distributed, naturally occurring phenolic acid, which is frequently found in commonly consumed products of plant origin such as onion, plum, grapes, nuts and spices [23]. In lamb’s lettuce protocatechuic acid was detected in very low concentrations compared with other phenolic compounds.

#### 3.1.2. Hydroxycinnamic Acids and Derivatives

Chlorogenic (5-*O*-caffeoylquinic acid) (compound **6**), caffeic (compound **9**) and *p*-coumaric (compound **15**) acids were directly identified by comparing their retention times with those of their corresponding standards and confirmed by MS/MS experiments. In the mass spectrum of compounds **9** and **15**, characteristic m/z values of 135 and 119, respectively, were observed, indicating the loss of CO_2_. In addition to 5-*O*-caffeoylquinic acid, the presence of another three caffeoylquinic acid isomers (compounds **2**, **7** and **10**) was confirmed by the loss of 162 Da (caffeic acid units) and their characteristic product ion patterns. Compound **2** was identified as neochlorogenic (3-*O*-caffeoylquinic acid) due to its relative retention time, its base peak at m/z 191 (quinic) and the intensity of fragment ions at m/z 179 and 135 [24]. Compound **7** was identified as cryptochlorogenic (4-*O*-caffeoylquinic acid) according to its base peak m/z 173 [quinic–H–H_2_O]^−^ and the typical less abundant fragment ions m/z 179, 191 and 135. Compound **10** was tentatively identified as cis-5-caffeoylquinic acid according to its retention time and fragmentation pattern, which was identical to 5-caffeoylquinic acid [25]. In agreement with previous studies in the study by V. locusta [18], chlorogenic acid was the principal phenolic compound found, to account, in our case, for 57.1% of the total phenolic concentration. Similarly to lamb’s lettuce, both lettuce and escarole have a high chlorogenic acid content, but in both the main hidroxycinnamic acid derivatives are *O*-caffeoylmalic acid and dicaffeoyltartaric acid [11]. Chlorogenic acid is a major phenolic compound in the leaves of other plant species, such as some Ericacea species [26] and many herbs [27]. Chlorogenic acid is one of the main polyphenols in the human diet, and it has been reported to have a variety of beneficial effects: for example, antioxidant [28], antidiabetic [29], antihypertensive [30] and anticancer [31] activities. It has been proposed as a nutraceutical for the prevention and treatment of the metabolic syndrome and associated disorders and as a food additive due to its potential to prevent the degradation of other bioactive compounds, and its prebiotic activity in humans [27].

Three compounds with fragment ions at m/z 341 (compounds **3**, **4** and **5**) were identified as caffeic acid *O*-hexoside derivatives and their identities were confirmed by a neutral loss scan of 162 Da and precursor scan experiments of 179 (caffeic). In addition, a product ion experiment revealed the characteristic loss of CO_2_ (m/z 135). As previously described, the glycosides eluted before their aglycone (caffeic acid) [32]. Although these and other caffeic derivatives are the main polyphenols in green leafy vegetables [11], they were found at relatively low concentrations in lamb’s lettuce.

The presence of three dicaffeoylquinic acids (compounds **23**, **24** and **28**) was confirmed by a parent ion at m/z 515 and a main product ion at m/z 353 (−162 Da, loss of caffeoyl moiety). The elution order of these compounds and the relative abundance of fragments at m/z 191, 179 173 and 135 led us to tentatively identify the compounds as 3,4-dicaffeoylquinic, 3,5-dicaffeoylquinic and 4,5-dicaffeoylquinic acids [25]. After chlorogenic acid, dicaffeoylquinic was the most abundant caffeoyl derivative in lamb’s lettuce. It is also present in lettuce [33] and in wild rosemary (*Eriocephalus africanus* L.), in which mono- and dicaffeolquinic acids were seen to represent 90% and 74%, respectively, of the total phenolics [34].

The product scan MS mode was used to monitor the fragmentation patterns of the ions with m/z 337 for coumaroylquinic (compounds **12** and **14**) and m/z 367 for feruloylquinic (compounds **13** and **16**) acids. All these hydroxycinnamoylquinic acids produce an intense ion at m/z 191 [quinic acid−H]^−^. Coumaroyl quinic acids also showed fragment ions at m/z 173 (loss of H_2_O) and 163 (loss of coumaroyl moiety). As both isomers (compounds **12** and **14**) showed identical fragmentation patterns, they were identified as cis 5-*O*-p-coumaroylquinic and trans 5-*O*-p-coumaroylquinic acids, according to the mass spectral characteristics reported by Baeza et al. [25]. Similarly, feruloylquinic acids (compounds **13** and **16**) presented mass spectral characteristic compatible with the isomer 5-*O*-p-feruoylquinic acid [25]. Once again, both compounds showed identical fragmentation so that they were tentatively identified as trans and cis 5-*O*-p-feruloylquinic acids. Coumaroylquinic acid was the second most common phenolic compound in lamb’s lettuce (9.7% of the total). It has also been found in lettuce [33], broccoli [35] and in several aromatic herbs [36]. Feruloylquinic acid was the fifth-ranked major compound in lamb’s lettuce. It can be found in a number of fruits such as blackcurrant, apricot, peach and plum [37], but few references have been found for leafy vegetables. In particular, feruloylquinic acid has been found Cichorium endivia [38] and recently it has been reported in *Artemisia annua* L. leaves [39].

Compound **31** was identified as feruloyl-caffeoylquinic acid based on its precursor ion at m/z 529, the base compound at m/z 353, produced by the loss of the feruloyl unit, and another intense ion at m/z 367 resulting from the loss of a caffeoyl unit [40]. This compound was found in lamb’s lettuce at low concentration.

Two compounds (compounds **8** and **11**) with an ion mass signal at m/z 385 were detected and identified as sinapic acid-hexoside according to their MS spectrum and previous data described by other authors in tronchuda cabbage [41]. In both compounds, the main fragment was found at m/z 223 (sinapic acid). In addition, minor product ions were observed at m/z 265 (loss of a part of hexose ring), 208 (methyl radical loss), 179 (decarboxylation), and 164 (combined methyl radical loss and decarboxylation). Sinapic acid and its derivatives have not received so much attention as other hydroxycinnamic acids, but their antioxidant and antibacterial effects are interesting for their application as natural food preservatives and for developing functional foods [42].

#### 3.1.3. Flavones

The aglycone luteolin (compound **34**) was identified by comparing its mass spectrum with that of the standard, which presented the expected fragmentation patterns with a precursor ion at m/z 285 and a characteristic fragment ion at m/z 133 and other fragment ions at m/z 175 and 151. Luteolin−7-*O*-glucoside (compound **21**) was identified by comparison of its retention time and mass spectrum with those of the corresponding standard. In addition, two more luteolin derivatives (compounds **17** and **18**) were detected and tentatively identified as luteolin−7-*O*-apiosylglucoside ([M − H]^−^ at m/z 579) and luteolin-7-rutinoside ([M − H]^−^ at m/z 593), both presenting the main fragment ion at m/z 285 (luteolin). Their identities were confirmed by their precursor ion spectra and neutral losses of 294 Da (162  +  132 Da) (apiosylglucoside moiety), 308 Da (146 + 162 Da) (rhamnosylglucoside moiety) and 162 Da (hexoside moiety) for compounds **17**, **18** and **21**, respectively. Luteolin is a flavone that usually occurs in its glycosylated forms in camomile and other species belonging to the Asteraceae family among others [43]. In lamb’s lettuce (thepresent study), luteolin-7-rutinoside was the main flavonoid found (4.3% of total phenolics). Several epidemiological studies have shown that luteolin possesses antioxidant, anti-inflammatory, antimicrobial and anticancer activities [43]. Luteolin-7-rutinoside has been previously identified in lettuce [11] and Mentha piperita [44] and luteolin-7-*O*-apiosylglucoside in celery [45].

The identification of compound **35** (diosmetin) and compound **30** (diosmin) was confirmed by comparing their retention times and mass spectra with their standards. Diosmetin presented the base compound ion at m/z 284 as a result of the loss of a methyl unit and further fragment ion at m/z 256 [M − H − CH_3_ − CO]^−^. Diosmin showed the base peak ion at m/z 299 as a result of the loss of 308 Da (162 + 146). Compound **27** exhibited the [M − H]^−^ ion at m/z 593, and a neutral loss of 294 Da (apiosylglucoside moiety), yielding fragment ions at m/z 299 (diosmetin), so that it was identified as diosmetin-apiosylglucoside. Diosmetin and its derivatives are mainly found in citrus fruits [46] but they have also been identified in parsley [47]. In lamb’s lettuce, the major forms were diosmin and diosmetin-apiosylglucoside. Diosmetin and its derivatives have been seen to possess potential biological activity with anticancer anti-inflammatory, antioxidant, antimicrobial and oestrogenic activities [48].

Other flavones found in lamb’s lettuce were two apigenin derivatives: by comparison with its standard, compound **29** with [M − H]^−^ ion at m/z 431 and a fragment at m/z 269 (resulting from the loss of a glucoside moiety) identified as apigenin−7-*O*-glucoside, while compound **25** with [M − H]^−^ ion at m/z 577 was identified as apigenin-rutinoside, since it exhibited the fragment at m/z 269, which is related to the loss of 308 Da (162 + 146). Apigenin−7-*O*-glucoside has been found as major polyphenol in chamomile flowers [49], while apigenin-rutinoside has been isolated from *Mentha longifolia* L. for use as condiment and a herbal tea [50]. In lamb’s lettuce, apigenin derivatives were found as minor polyphenolic compounds.

The precursor ion of compound **32** was detected at m/z 591 and a characteristic MS/MS fragment ion at m/z 283 (−308 Da), so it was tentatively identified as acacetin-rutinoside. This flavone has been identified in Compositae species [51].

#### 3.1.4. Flavonols

Quercetin (compound **33**) was directly identified by comparison of its retention time with the corresponding standard and confirmed by MS/MS experiments. It showed a precursor ion at m/z 301, a characteristic fragment ion at m/z 151 and other fragment ions at m/z 179, 121 and 107. As regards quercetin derivatives, compound **20** was identified as quercetin-glucuronide with an [M − H]^−^ ion at m/z 477 and the main fragment ions at m/z 301 due to the loss of a glucuronyl (176 Da) unit. Compound **22** with an [M − H]^−^ ion at m/z 463, presented two high intensity fragments at m/z 301 [M − H − 162]^−^ and 300 [M − H − 162]^−•^. The radical aglycone was the most abundant fragment for collision energies from 5 to 30 eV and presented similar abundance to that of the aglycone at higher collision energies. The higher intensity of the radical aglycone compared with the aglycone suggested 3-OH was the glycosylation site [52], so this compound was attributed to quercetin−3-*O*-glucoside. The concentrations of both quercetin and its derivatives were low in lamb’s lettuce compared to other polyphenols.

Compound **19** was identified as isorhamnetin-rutinoside with an [M − H]^−^ ion at m/z 623 and a characteristic product ion at m/z 315 corresponding to isorhamnetin aglycone and a loss of 308 Da (rutinose). This compound is commonly extracted from marigold for medicinal purposes (*Calendula officinalis* L.) [53] but is also found in other vegetables such as Asparagus acutifolius [54].

#### 3.1.5. Flavanones

The mass spectral characteristics of compound **26**, with [M − H]^−^ at m/z 609 and the main fragment at m/z 301 as a result of the loss of a rutinoside moiety (−308 Da), corresponded to hesperetin−7-*O*-rutinoside (hesperidin), which is a common flavanone in citrus fruits [55] but the only one we detected in lamb’s lettuce.

### 3.2. Carotenoid and Chlorophyll Profiling

#### 3.2.1. Carotenoids

The chromatographic behavior and UV-Vis absorption spectrum allowed identification of seven xanthophyll and four carotene pigments in lamb’s lettuce leaves (Table 2). All-trans-violaxanthin (compound **1**), 9 cis-neoxanthin (compound **2**), antheraxanthin (compound **4**), all-trans-lutein (compound **7**), zeaxanthin (compound **8**) and β-cryptoxanthin (compound **12**) were identified based on a comparison of their retention times and spectra with those of the corresponding standard. Taking into consideration its chromatographic and spectroscopic properties, compound **3** was identified as luteoxanthin [56]. In agreement with the results reported for other leafy vegetables, lutein was the major xanthophyll found in lamb’s lettuce (28% of total carotenoid content), with values in the range of those described for different types of lettuce [57]. As expected for a green leafy vegetable, neoxanthin was found in the 9 or 9′ cis isomer form [58] since this isomer is present in the chloroplasts, while the all-trans-neoxanthin is found only in non-photosynthetic organs [59]. However, contrarily to the results reported for other species [57], the neoxanthin concentration was higher than that of violaxanthin. Minor xanthophylls such as zeaxanthin and β-cryptoxanthin were present in concentrations well over the values reported for other leafy vegetables [60]. Antheraxanthin has previously been found in commonly consumed leafy vegetables such as spinach [61], chicory, dandelion, garden rocket wild rocket [62]. The presence of luteoxanthin has been reported in spinach [63] and medical herbs [1], but our study identified it, for the first time, in lamb’s lettuce. The role of xanthophylls in vision health has been extensively studied. Zeaxanthin and lutein, particularly, play an important role in photoprotection against macular degeneration and there is also evidence that zeaxanthin and lutein play a role in visual and auditory processing, general mental acuity, and protection against various chronic diseases [64].

The identification of both all-trans-β-carotene and all-trans-α-carotene was based on the use of their standards. In the case of cis-isomers, their spectral fine structure and peak cis intensity were considered (Table 2). Compounds **13** and **18** were identified as 13-cis- and 9-cis-β-carotene, respectively, according to their order of elution, the hypsochromic shift of 9 and 5 nm, and the Q-ratios similar to those previously reported [65,66]. Carotenes represented 66% of the total carotenoid content, all-trans-β-carotene being the major carotene (accounting for 58% of total carotenoids), as previously has been previously reported for lamb’s lettuce and other leafy vegetables [67]. Among carotenes, β-carotene exhibits the highest pro-vitamin A potential, although α-carotene and β-cryptoxanthin play a similar role. In addition to many other fundamental functions in human health, β-carotene helps prevent the progression of eye diseases by quenching free radicals and thus attenuating oxidative stress [68].

#### 3.2.2. Chlorophylls

Chromatographic analysis with two serially coupled C_30_ columns allowed the simultaneous separation of chlorophylls and carotenoids (Table 2). According to their characteristic UV-Vis spectra, compounds **5** and **9** corresponded to chlorophylls a and b, respectively [69]. In additions, compounds **6** and **10** were identified as chlorophylls a’ and b’. These two chlorophyll epimeric isomers have identical absorption spectra to those of chlorophylls a and b, which eluted before their corresponding epimers due to their higher polarity [70]. Finally, the absorbance spectra and the chromatographic behavior of compounds **15** and **17** were in agreement with those reported for pheophytin a and pheophytin a’ [70].

As the role of carotenoids as bioactive compounds has been widely investigated, studies on chlorophylls are relatively scarce. There have been some reports on the antioxidant capacity of chlorophylls [71,72]. Ferruzzi et al. [8] have suggested that chlorophylls may play a role in human health and disease prevention. Indeed, the potential bioactivity of dietary chlorophyll derivatives with antioxidant and antimutagenic activities has been suggested. The antioxidant action of chlorophyll has been observed in vivo, providing protection to the liver and kidneys from the oxidative stress caused by sodium nitrate [73]. However, compared with carotenoids, little is known about chlorophyll metabolites, their absorption, transport, metabolic pathways and their oxidation mechanisms [72], and more studies are needed.

### 3.3. Effect of Mineral Nutrition and Salinity on Lamb’s Lettuce Composition

In order to know the effect of mineral nutrition on the different phenolic compounds, they were grouped into phenolic families. The calcium (Ca) concentration and salinity of the nutrient solution had no effect on the main phenolic families (Table 3), while potassium (K) and nitrogen (N) levels had a significant effect on most of the families. The lowest K concentration (0.1 mM) significantly increased flavone (37%), and flavanone (46%) concentrations compared to treatment with 3.5 mM K. The flavanone content was also significantly higher (45%) in the 0.5 mM K treatment than in the presence of the highest K concentration. Reducing the concentration of N from 7 mM to 1 or 0.1 mM had a similar effect, leading to significantly higher hydroxycinnamic acid (40–48%), flavonol (40–44%) and flavanone (2.6–3.6-fold) concentrations. In addition, treatment with 0.1 mM N increased the flavone content to a greater extent (3.5-fold) than 1 mM N (2.3-fold). In agreement with the results for lamb’s lettuce, a K deficiency in spinach increased total phenolic and flavonoid contents in non-saline conditions [74]. Similarly, in lettuce and other leafy vegetables, previous studies have also shown that a reduction in the N supply enhances the phenolic content and antioxidant capacity [75,76]. Moreover, the extracts from lettuce plants grown under low nitrogen conditions had a more pronounced anti-proliferative effect on colorectal cancer than those from lettuce grown with an adequate nitrogen supply, which was attributed to enhanced phenolic concentrations [77]. For this reason, the authors suggested that vegetables with improved health-related properties could be developed by increasing the phenolic content through a reduction in nitrogen nutrition.

The carotenoid content of lamb’s lettuce was significantly affected by K and N levels and salinity, but not by Ca (Table 4). The lowest level of K (0.1 mM) significantly increased total carotenoids (82%), mainly as a result of the increase in *β*-carotene and lutein, the major carotenoids identified in the present work (data not shown). Similar results in relation to K have been found in spinach, a deficiency increasing the carotenoid and flavonoid contents, as mentioned [74]. By contrast, low N (0.1 and 1 mM) concentrations led to a lower total carotenoids content. In spinach, N deficiency enhanced the phenolic and anthocyanin contents but drastically reduced the carotenoid content [74]. As regards the effect of salinity, only the highest concentration of NaCl (60 mM) led to a significant increase of 58% in total carotenoids. A similar increase in carotenoids under salinity has been found in spinach [74].

The total chlorophyll content increased as the concentration of Ca and K in the nutrient solution decreased (Table 4). Contrarily to Ca and K, low levels of N and the highest level of salinity (60 mM NaCl) lowered the chlorophyll content. A decrease in N levels also decreased the total chlorophyll content of lamb’s lettuce cv. Princess [78] and similarly, in spinach [79]

Lamb’s lettuce was seen to be a good source of vitamin C with similar or even higher values than those reported for other commonly consumed leafy vegetables such as lettuce or spinach [54]. Its content in lamb’s lettuce was not affected by the reduction in any of the studied plant mineral nutrients. However, in other salad species, including lettuce, changes in ascorbic acid have been related with mineral nutrition [80]. Regarding salinity, the highest concentration of NaCl (60 mM) significantly increased the vitamin C content, as has been found in *Amaranthus* leafy vegetables [81] and tomato fruits [82]. Our results agree with those of El-Nakel et al. [80], who indicated that nutritional chemical stress (e.g., mild to moderate salinity and nutrient stress) can improve the nutritional value of vegetables through the accumulation of certain metabolites as a response in their adaptation to suboptimal conditions.

## 4. Conclusions

Chromatographic analysis of the phenolic profile revealed the presence of 35 phenolic compounds in lamb’s lettuce. The main compounds were chlorogenic, coumaroylquinic, dicaffeoylquinic and feruloylquinic acids, and luteolin-7-rutinoside, disometin-apiosylglucoside, diosmin and sinapic acid-hexoside. The major carotenoids identified were *β*-carotene and lutein. According to our results, lamb’s lettuce can be considered a good option as a salad ingredient due to its phenolic, carotenoid, chlorophyll and vitamin C content. Many of the identified secondary metabolites are reported here in lamb’s lettuce for the first time. Variations in the concentrations of some of these compounds were observed as a result of different fertilization doses and salinity levels. Low levels of K increased flavones, flavanones, carotenoids and chlorophylls, while a reduction in the N concentration led to an even greater increase in all the phenolic families but reduced the carotenoid and chlorophyll content. Finally, salinity increased the carotenoid and vitamin C contents, but decreased that of chlorophylls. These results highlight the impact of plant mineral nutrition on the accumulation of bioactive compounds and point to the management of fertilization or saline conditions as a useful tool for increasing the phytochemical content and functional quality of lamb’s lettuce. More studies are needed to explore the impact of other genetic (cultivar) and preharvest factors on secondary metabolite content as future strategies to improve the functional value of lamb’s lettuce.

## Figures and Tables

**Table 1:** Phenolic compounds identified in lamb’s lettuce by MS/MS approaches. Retention time (RT, min), precursor ion ([M − H]^−^), base peak (100% relative abundance) (bp) and other fragments (and their relative abundances) detected in the product ion mode and concentration (C) of each compound (µg g^−1^ fresh weight).

	Compound	RT	[M − H]^−^	bp	Product Ions	C ^a^
**1**	Protocatechuic	11.65	153	109		0.015
**2**	3-Caffeoylquinic acid	12.31	353	191	179(55), 135(6)	0.15
**3**	Caffeic acid-*O*-hexoside 1	14.28	341	179	135(10)	0.093
**4**	Caffeic acid-*O*-hexoside 2	16.71	341	179	135(4)	0.036
**5**	Caffeic acid-*O*-hexoside 3	17.33	341	179	135(14)	0.011
**6**	5-Caffeoylquinic acid	18.06	353	191	179(2), 173(1)	367.6
**7**	4-Caffeoylquinic acid	18.46	353	173	191(55), 179(85), 135(40)	3.2
**8**	Sinapic acid-hexoside 1	19.18	385	223	208(3), 179(5), 164(5)	7.4
**9**	Caffeic acid	20.47	179	135		2.3
**10**	*cis*-5-Caffeoylquinic acid	20.96	353	191	179(7), 173(1)	55.5
**11**	Sinapic acid-hexoside 2	21.89	385	223	208(5), 179(3), 164(2)	5.2
**12**	*cis* 5-*O*-*p*-Coumaroylquinic	23.48	337	191	173(6), 163(4)	35.1
**13**	*trans* 5-*O*-*p*-Feruloylquinic acid	25.73	367	191	173(8)	20.1
**14**	*trans* 5-*O*-*p*-Coumaroylquinic acid	25.93	337	191	173(1), 163(1)	27.1
**15**	*p*-Coumaric acid	27.53	163	119		0.056
**16**	*cis* 5-*O*-*p*-Feruloylquinic acid	28.01	367	191	173(3)	10.0
**17**	Luteolin-7-*O*-apiosylglucoside	32.80	579	285		1.2
**18**	Luteolin-7-rutinoside	33.72	593	285		27.9
**19**	Isorhamnetin-rutinoside	34.18	623	315		1.5
**20**	Quercetin-glucuronide	34.51	477	301		0.01
**21**	Luteolin-7-*O*-glucoside	34.99	447	285		0.20
**22**	Quercetin-3-*O*-glucoside	35.09	463	300	301(35)	0.031
**23**	3,4-Dicaffeoylquinic acid	35.98	515	353	191(6), 179(5), 173(2)	3.1
**24**	3,5-Dicaffeoylquinic acid	37.61	515	353	191(5), 179(4)	26.1
**25**	Apigenin-rutinoside	38.12	577	269		0.25
**26**	Hesperidin	38.48	609	301		1.1
**27**	Diosmetin-apiosylglucoside	39.75	593	299	284(1)	23.9
**28**	4,5-Dicaffeoylquinic acid	40.50	515		191(15), 179(50), 173(65)	1.5
**29**	Apigenin-7-*O*-glucoside	40.69	431	269		0.012
**30**	Diosmin	40.80	607	299	284(1)	21.6
**31**	Feruloyl-caffeoylquinic acid	43.91	529	353	367(55), 191(10), 179(17)	0.043
**32**	Acacetin-rutinoside	50.00	591	283		1.1
**33**	Quercetin	50.99	301	151	179(24), 121(42), 107(33)	0.15
**34**	Luteolin	51.14	285	133	175(10), 151(10)	0.051
**35**	Diosmetin	54.78	299	284	256(11)	0.16

^a^ Mean values of plants (edible part) grown in standard conditions (½ Hoagland).

**Table 2:** Tentative identification, retention time (RT, min) spectral characteristic (absorbance maxima and Q-ratios found in the present study and those reported in the literature) and concentration (C, µg g^−1^ fresh weight) of carotenoids and chlorophylls in lamb’s lettuce. Wavelengths given in parenthesis denote shoulders.

	Compound	RT			λ (nm)			Q_ratio_ Found	Q_ratio_ Reported	C ^a^
**1**	all-*trans-*violaxanthin	10.68			416	440	468			5.5
**2**	9 or 9′-*cis*-neoxanthin	11.58		328	412	436	464	0.11	0.13 [56]	8.1
**3**	luteoxanthin	12.44			398	422	448			3.3
**4**	antheraxanthin	14.72			422	444	472			3.8
**5**	chlorophyll b	15.42			468	602	652			
**6**	chlorophyll b’	17.26			468	602	652			
**7**	all-*trans-*lutein	17.96			(422)	444	472			27.2
**8**	zeaxanthin	22.24			(428)	450	478			0.62
**9**	chlorophyll a	23.45			432	618	666			
**10**	chlorophyll a’	27.09			432	618	666			
**11**	*β*-apo-8′-carotenal ^b^	28.38				466				
**12**	*β*-cryptoxanthin	38.52			(426)	452	478			0.41
**13**	13-*cis*-*β*-carotene	44.59		338	(424)	446	470	0.39	0.35 [62]	1.9
**14**	all-*trans*-*α*-carotene	47.55			(426)	446	474			1.2
**15**	pheophytin a	53.22	408	506	538	610	666			
**16**	all-*trans*-*β*-carotene	54.93			(428)	452	478			57.4
**17**	pheophytin a’	56.85	408	506	538	610	666			
**18**	9-*cis*-*β*-carotene	59.81		342	(426)	446	474	0.08	0.10 [63]	3.2

^a^ Mean values of plants (edible part) grown in standard conditions (½ Hoagland). ^b^ Internal standard.

**Table 3:** Concentration of main phenolic families (µg g^−1^ fresh weight) in lamb’s lettuce under different nutritional conditions. Values are means ± SE (*n* = 4).

	mM	Hydroxicinamic	Flavones	Flavonols	Flavanones
Ca	0.5	838 ± 90	113 ± 9	3.54 ± 0.54	4.27 ± 0.67
	2	771 ± 12	119 ± 7	3.96 ± 0.21	3.70 ± 0.12
	5	779 ± 20	130 ± 9	4.50 ± 0.48	4.90 ± 0.31
		n.s.	n.s.	n.s.	n.s.
	0.1	302 ± 49	44 ± 1 ^b^	0.46 ± 0.15	1.66 ± 0.13 ^b^
K	0.5	259 ± 17	37 ± 2 ^a^	0.21 ± 0.10	1.64 ± 0.20 ^b^
	3.5	235 ± 29	32 ± 1 ^a^	0.30 ± 0.24	1.13 ± 0.12 ^a^
		n.s.	**	n.s.	*
N	0.1	840 ± 18 ^b^	263 ± 4 ^c^	2.32 ± 0.16 ^b^	3.09 ± 0.26 ^b^
	1	791 ± 85 ^b^	173 ± 29 ^b^	2.38 ± 0.24 ^b^	3.73 ± 0.67 ^b^
	7	565 ± 12 ^a^	76 ± 4 ^a^	1.65 ± 0.14 ^a^	1.05 ± 0.06 ^a^
		**	***	*	**
NaCl	C	479 ± 16	63 ± 2	2.10 ± 0.18	0.92 ± 0.12
	15	471 ± 25	60 ± 2	1.99 ± 0.32	0.87 ± 0.07
	30	480 ± 56	52 ± 2	1.87 ± 0.34	0.74 ± 0.05
	60	397 ± 116	46 ± 13	2.19 ± 0.41	0.75 ± 0.19
		n.s.	n.s.	n.s.	n.s.

*, **, *** Significant differences between means at 5, 1 or 0.1% level of probability, respectively; n.s., non-significant at *p* = 5%. For each stage, different letters in the same column indicate significant differences between means according to Duncan’s test at the 5% level.

**Table 4:** Concentration of vitamin C, *β*-carotene and lutein (µg g^−1^ fresh weight) in lamb’s lettuce grown under different nutritional conditions. Values are means ± SE (*n* = 4).

Treatments	mM	Total Carotenoids	Total Chlorophyll	Vitamin C
Ca	0.5	130 ± 10	125 ± 8 ^b^	376 ± 4
	2	150 ± 7	86 ± 5 ^a^	364 ± 6
	5	154 ± 18	89 ± 9 ^a^	356 ± 10
		n.s.	**	n.s.
K	0.1	148 ± 16 ^b^	205 ± 22 ^b^	454 ± 8
	0.5	100 ± 8 ^a^	140 ± 13 ^a^	397 ± 21
	3.5	82 ± 4 ^a^	114 ± 7 ^a^	425 ± 26
		**	**	n.s.
N	0.1	91 ± 4 ^a^	94 ± 3 ^a^	661 ± 20
	1	86 ± 9 ^a^	87 ±8 ^a^	682 ± 55
	7	109 ± 3 ^b^	116 ±3 ^b^	624 ± 3
		*	**	n.s
NaCl	0	83 ± 3 ^a^	100 ± 4 ^b^	515 ± 10 ^a^
	15	113 ± 7 ^ab^	101 ±6 ^b^	541 ± 17 ^ab^
	30	121 ± 8 ^ab^	88 ± 4 ^ab^	540 ± 20 ^ab^
	60	132 ± 16 ^b^	80 ± 1 ^a^	597 ± 20 ^b^
		*		*

*, ** Significant differences between means at 5, 1% level of probability, respectively; n.s., non-significant at *p* = 5%. For each stage, different letters in the same column indicate significant differences between means according to Duncan’s test at the 5% level.

## Data Availability

The data presented in this study are available on request from the corresponding author.
